# Variations in the Hemagglutinin of the 2009 H1N1 Pandemic Virus: Potential for Strains with Altered Virulence Phenotype?

**DOI:** 10.1371/journal.ppat.1001145

**Published:** 2010-10-14

**Authors:** Jianqiang Ye, Erin M. Sorrell, Yibin Cai, Hongxia Shao, Kemin Xu, Lindomar Pena, Danielle Hickman, Haichen Song, Matthew Angel, Rafael A. Medina, Balaji Manicassamy, Adolfo Garcia-Sastre, Daniel R. Perez

**Affiliations:** 1 Virginia-Maryland Regional College of Veterinary Medicine, College Park, Maryland, United States of America; 2 Department of Veterinary Medicine, University of Maryland, College Park, Maryland, United States of America; 3 Synbiotics Corporation, College Park, Maryland, United States of America; 4 Department of Microbiology, Mount Sinai School of Medicine, New York, New York, United States of America; 5 Institute of Global Health and Emerging Pathogens, Mount Sinai School of Medicine, New York, New York, United States of America; 6 Department of Medicine, Division of Infectious Diseases, Mount Sinai School of Medicine, New York, New York, United States of America; University of California San Francisco, United States of America

## Abstract

A novel, swine-origin influenza H1N1 virus (H1N1pdm) caused the first pandemic of the 21^st^ century. This pandemic, although efficient in transmission, is mild in virulence. This atypical mild pandemic season has raised concerns regarding the potential of this virus to acquire additional virulence markers either through further adaptation or possibly by immune pressure in the human host. Using the mouse model we generated, within a single round of infection with A/California/04/09/H1N1 (Ca/04), a virus lethal in mice—herein referred to as mouse-adapted Ca/04 (ma-Ca/04). Five amino acid substitutions were found in the genome of ma-Ca/04: 3 in HA (D131E, S186P and A198E), 1 in PA (E298K) and 1 in NP (D101G). Reverse genetics analyses of these mutations indicate that all five mutations from ma-Ca/04 contributed to the lethal phenotype; however, the D131E and S186P mutations—which are also found in the 1918 and seasonal H1N1 viruses—in HA alone were sufficient to confer virulence of Ca/04 in mice. HI assays against H1N1pdm demonstrate that the D131E and S186P mutations caused minor antigenic changes and, likely, affected receptor binding. The rapid selection of ma-Ca/04 in mice suggests that a virus containing this constellation of amino acids might have already been present in Ca/04, likely as minor quasispecies.

## Author Summary

The 19^th^ century experienced three major influenza pandemics: the Spanish flu of 1918 (H1N1), the Asian flu of 1957 (H2N2) and the Hong Kong flu of 1968 (H3N2). These pandemics were introduced into the human population through the accumulation of avian and human influenza genes (genetic reassortment), creating with each pandemic a novel influenza virus, one that the human population had not been exposed to. These pandemics were associated with high morbidity and mortality; the 1918 pandemic was responsible for an estimated 40 million deaths. In April 2009, a novel, swine-origin influenza H1N1 virus (H1N1pdm) caused the first influenza pandemic of the 21^st^ century. This pandemic was a result of genetic reassortment between not only human and avian influenza genes, but also swine influenza genes. This H1N1 pandemic, although efficient in human-to-human transmission, differed greatly from the previous pandemics in its mild virulence. This atypical mild pandemic season has raised concerns regarding the potential of this virus to become more virulent. Using a mouse model, we were able to demonstrate that this pandemic strain is amenable to mutations that lead to a more virulent virus.

## Introduction

In April 2009, a novel H1N1 influenza virus (H1N1pdm) emerged in North America causing the first influenza pandemic of the 21^st^ century [1], [2], [3]. This pandemic strain is a triple-reassortant virus of swine origin containing genes from avian, swine and human influenza viruses. It is genetically related to triple reassortant swine H1N1 influenza viruses currently circulating in North America, with the exception of the neuraminidase (NA) and matrix (M) genes, which derive from an Eurasian swine influenza virus. Although the virus is highly transmissible in humans, H1N1pdm infections have been characterized with mostly mild symptoms [1], [2], [3]. In addition to some preexisting immunity [4], the relatively mild illness associated with H1N1pdm in humans is consistent with the absence of several virulence markers previously identified in other human and avian influenza virus strains. The H1N1pdm lacks K627 in the polymerase basic protein 2 (PB2), does not encode the pro-apoptotic PB1-F2 protein, and contains a nonstructural NS1 protein lacking the PDZ domain [5], [6], [7]. Interestingly, reports indicate that introducing K627 into the PB2 or the PB1F2 protein into the PB1 gene does little to enhance the pdm H1N1's virulence [8], [9]. The H1N1pdm's mild presentation of disease resembles the first wave of the 1918 and 1968 pandemics which were followed by a more severe second wave [10]. In swine, the triple reassortant influenza viruses have shown a tendency to reassort and give rise to viruses with distinct antigenic characteristics [11]. Thus, the potential acquisition of virulence markers by the H1N1pdm virus is of great concern.

To date there are no conclusive reports that indicate an increase in virulence of the H1N1pdm virus, although some severe cases have been associated with a D222G mutation on the HA [12], [13], [14], [15], [16]. Therefore, we wanted to determine if the H1N1pdm virus could gain virulence through adaptation in the mouse model. In our initial studies with the prototypical H1N1pdm strain, A/California/04/09 (H1N1), (Ca/04) we observed that the virus was not lethal for Balb/c mice at a dose of 10^6^ TCID_50_. However, DBA/J2 mice, which have recently been shown to be more susceptible to influenza virus infections than Balb/c mice [17], [18], succumbed to challenge with the same virus dose. Interestingly, when Balb/c mice were infected with lung homogenates from Ca/04-infected DBA/J2 mice, an increase in virulence was observed. Further testing of this virus showed a 1,000-fold increase in virulence in Balb/c as compared to wild type (wt) Ca/04 by 50% lethal dose (LD50) assays. Sequence and reverse genetics analyses identified 5 amino acid changes in the PA, HA and NP genes that were responsible for the lethality in Balb/c mice. Although all five mutations participated in modulating virulence, those in the HA were the most important. Of particular significance are the D131E and S186P mutations in HA, which are also found in the 1918 pandemic viruses, suggesting that the H1N1pdm could become further adapted in and/or virulent for humans.

## Results

### A virulent H1N1pdm virus is rapidly selected in DBA/J2 mice

**Figure 1:**
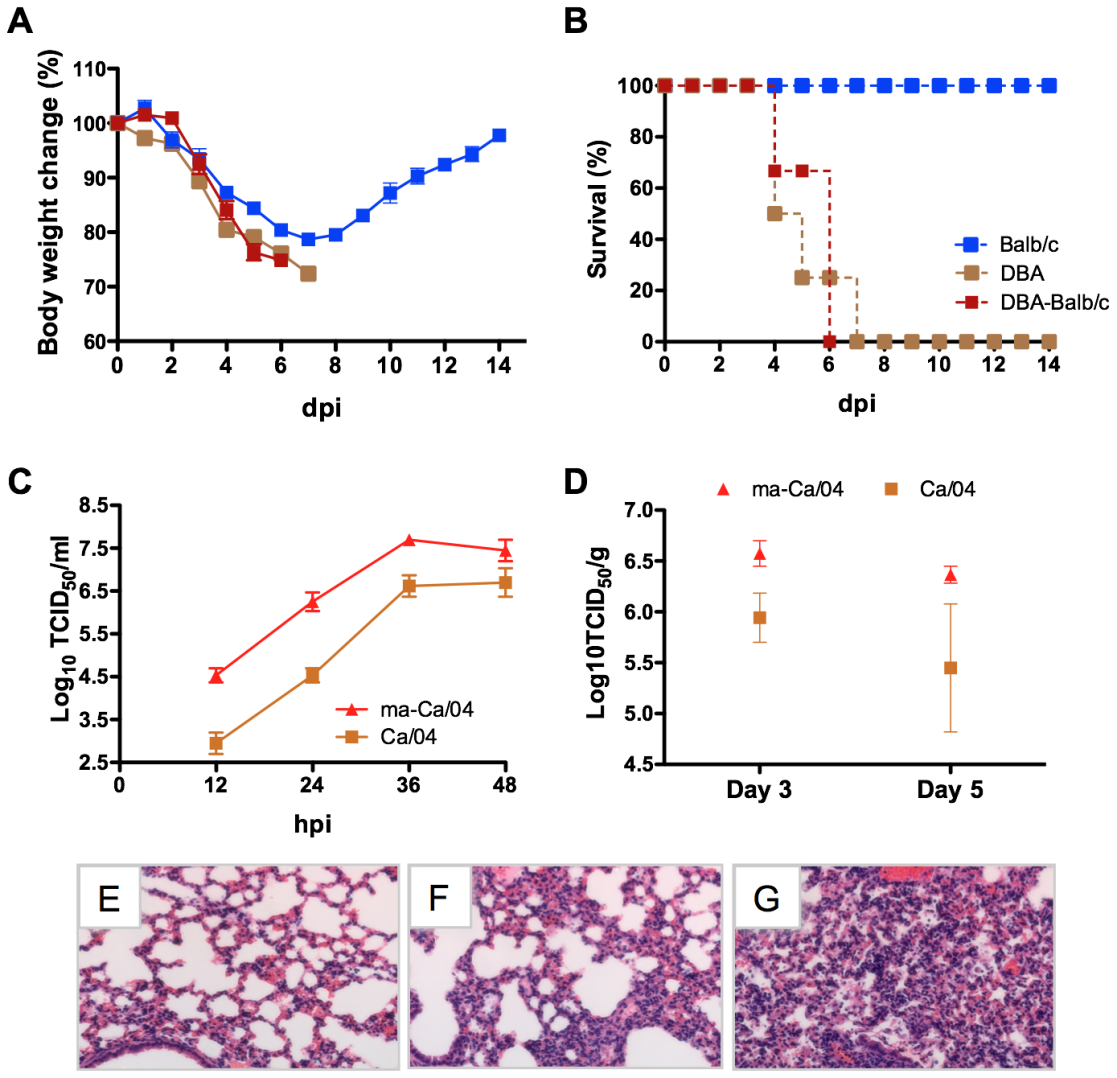
Generation of an H1N1pdm virus lethal for Balb/c mice. A) % Body weight changes and B) % survival of Balb/c (n = 4) and DBA (n = 4) mice inoculated intranasally with 5.4×10^5^ TCID_50_ of Ca/04. DBA-Balb/c corresponds to Balb/c mice infected with lung homogenates containing the first passage Ca/04 virus from DBA mice. C) Growth kinetics of Ca/04 and ma-Ca/04 in MDCK cells. Viruses were inoculated at a multiplicity of infection of 0.001. Supernatants were collected at the indicated time points and titrated in MDCK cells by TCID_50_. D) Balb/c mice inoculated intranasally with 1.2×10^4^ TCID_50_ of ma-Ca/04 or Ca/04. Lungs from infected mice (n = 3/time point) were collected at 3 and 6 dpi, homogenized and virus titers measured in MDCK cells by TCID_50_. Results are expressed as log_10_ TCID_50_/gr of tissue. E, F, G) Balb/c mouse lungs were collected at 3 dpi as described in D and fixed in 10% formalin, embedded in paraffin and sectioned. Serial sections were stained with H&E and the images were captured at ×20 magnification. E) Mock infected lung, F), Ca/04-infected lung, G) ma-Ca/04-infected lung.

Pathogenesis studies have been performed in mice and other animal models in order to elucidate the H1N1pdm virus's molecular markers of virulence [19], [20]. Consistent with previous reports, we observed that the H1N1pdm virus was virulent for Balb/c mice, however only at high concentrations (≥10^7^ TCID_50_). In our study, Balb/c mice infected intranasally with 5.4x10^5^ TCID_50_ of Ca/04 showed significant body weight loss (∼20%) during the first 7 days post-infection (dpi), but eventually recovered (Fig 1A, B). This is in stark contrast to the 1918 pandemic strains, which are lethal for mice at concentrations significantly lower than 10^5^ TCID_50_ [21]. In contrast to the Balb/c study, we found that Ca/04 was lethal for DBA/J2 mice, which succumbed to infection by 7 dpi at the same dose of 5.4×10^5^ TCID_50_ (Fig 1A, B). This result is not necessarily unexpected since previous studies have shown that DBA/J2 mice have an increased susceptibility to influenza virus infection [17], [18], [22]. In order to determine whether the virus population lethal to DBA/J2 mice had an altered phenotype compared to the Ca/04 virus used as inoculum, lung homogenates from the affected DBA/J2 mice were used to infect three naïve Balb/c mice (DBA-Balb/c, Fig 1A, B). Interestingly, the virus contained in the DBA-mouse lung homogenates was lethal for Balb/c mice (Fig 1A, B). Balb/c mice succumbed to infection by 7 dpi, a timeframe that resembled the Ca/04 infection of DBA mice. The virus titer of the DBA-lung homogenates, used as inoculum for infection of Balb/c mice, was 1.2×10^5^ TCID_50_, indicating that lethality in Balb/c mice was not due to an increased virus dose.

Because we could not rule out the possibility that the lethality of the virus present in the DBA-mouse lung homogenates was not due to the presence of cytokines and other substances toxic to Balb/c mice, lung homogenates from the Balb/c mice were grown in MDCK cells and the virus obtained was arbitrarily designated as mouse-adapted Ca/04 (ma-Ca/04). In vitro growth kinetics revealed that the ma-Ca/04 virus grew faster and yielded more than 10-fold higher titers than Ca/04 in MDCK cells (Fig 1C). To further characterize the ma-Ca/04 virus, pathogenesis and replication were evaluated in the Balb/c mouse and ferret models. The mouse 50% lethal dose (MLD_50_) of ma-Ca/04 in Balb/c mice was 3.3 (log_10_TCID_50_), whereas that of the wild type virus was >6.0 (log_10_TCID_50_) indicating that indeed the virus had become more virulent for Balb/c mice (Table 1). Since we use virus from MDCK tissue culture supernatants, it is unlikely that cytokines or other substances are contributing to the ma-Ca/04 more lethal phenotype. The ma-Ca/04 also grew to higher titers in the lungs of mice compared to Ca/04 by 3 dpi (Fig 1D). H&E staining showed that the Ca/04 caused mild and limited alveolitis (Fig 1F). In contrast, lungs of mice infected with the ma-Ca/04 showed prominent lung pathology with severe alveolitis, characterized by the infiltration of neutrophils, lymphocytes and macrophages (Fig 1G). Immunohistochemistry for viral antigen showed more extensive positive staining in the bronchiolar lumen of ma-Ca/04-infected lungs than in the Ca/04-infected lungs (Figure S1 A, B, D and E). We also observed focal antigen staining in the alveolar area of the ma-Ca/04-infected lungs (Figure S1 F), whereas virus antigen was hardly detected in the alveolar area of Ca/04-infected lungs (Figure S1 C). In summary, these studies suggest that the ma-Ca/04 has increased virulence in the Balb/c mouse model when compared to Ca/04.

**Table 1:** MLD_50_ of wt and recombinant H1N1pdm viruses in Balb/c mice.

Virus*	MLD_50_ (log_10_TCID_50_)
Ca/04	>6.0
ma-Ca/04	3.3
maHA1:7Ca/04	6.0
maHA-PA2:6Ca/04	4.6
maHA-NP2:6Ca/04	4.8
maHA-PA-NP3:5Ca/04	3.8
NL/602	6.0
NY/18HA1:7NL/602	>6.0
maHA1:7NL/602	3.4

*Ca/04, A/California/04/09 (H1N1); ma-Ca/04, mouse-adapted Ca/04; maHA1:7Ca/04, recombinant carrying the HA gene from ma-Ca/04 and remaining 7 genes from Ca/04; maHA-PA2:6Ca/04, recombinant carrying the HA and PA genes from ma-Ca/04 and remaining 6 genes from Ca/04; maHA-NP2:6Ca/04, recombinant carrying the HA and NP genes from ma-Ca/04 and remaining 6 genes from Ca/04; maHA-PA-NP3:5Ca/04, recombinant carrying the HA, PA, and NP genes from ma-Ca/04 and remaining 5 genes from Ca/04; NL/602, A/Netherlands/602/09 (H1N1); NY/18HA1:7NL/602, recombinant carrying the HA gene from A/New York/18/09 (H1N1) and remaining 7 genes from NL/602; maHA1:7NL/602, recombinant carrying the HA gene from ma-Ca/04 and remaining 7 genes from NL/602.

**Figure 2:**
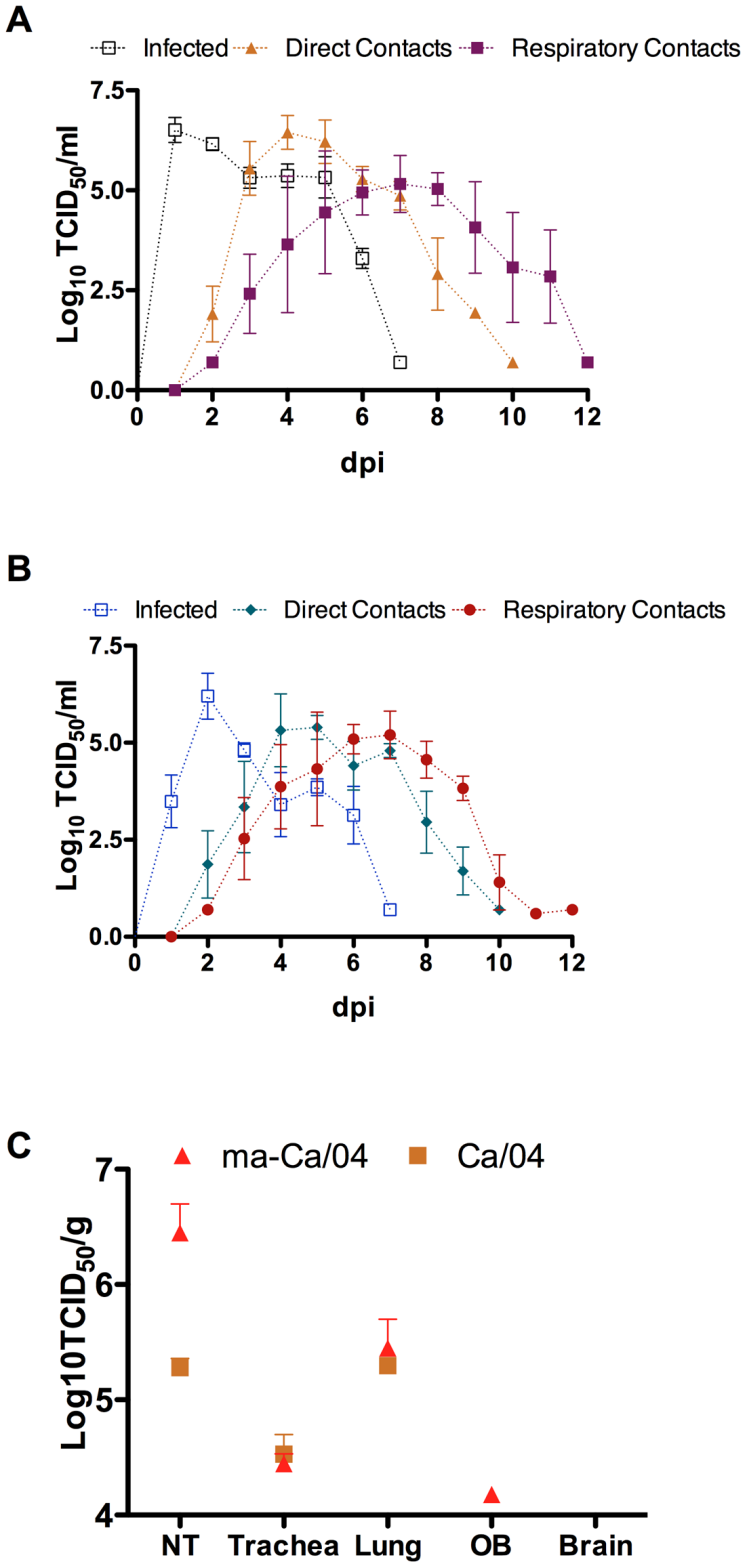
Replication and transmission of ma-Ca/04 in ferrets. A) Four groups of ferrets consisting of 1 infected, 1 direct contact (DC), and 1 respiratory contact (RC) per group were infected with 10^6^ TCID_50_ of ma-Ca/04 as described in materials and methods. The graph represents the average ± SD of virus shedding (log_10_ TCID_50_/ml of nasal wash) over time (in days, dpi) of inoculated (infected, grey open squares), direct contacts (orange triangles), and respiratory contact ferrets (purple squares). B) Two groups of ferrets consisting of 1 infected, 1 direct contact (DC), and 1 respiratory contact (RC) per group were infected with 10^6^ TCID_50_ of Ca/04 as described in materials and methods. The graph represents the average ± SD of virus shedding (log_10_ TCID_50_/ml of nasal wash) over time (in days, dpi) of inoculated (infected, blue open squares), direct contacts (green diamonds), and respiratory contact ferrets (red dots). Transmission was monitored by titrating the amount of virus in the nasal washes of ferrets collected daily. BLD, below limit of detection (0.699 log_10_ TCID_50_/ml). C) Two ferrets/group were infected with 10^6^ TCID_50_ of either Ca/04 or ma-Ca/04. Infected ferrets were euthanized at 4 dpi and brains, olfactory bulbs nasal turbinates, tracheas, and lungs were collected, homogenized and titrated in MDCK cells by TCID_50_. Results show log_10_ TCID_50_/gr of tissue.

To determine whether mouse adaptation of the Ca/04 virus resulted in an altered fitness for other mammalian species, we compared the replication and transmission of ma-Ca/04 to Ca/04 in ferrets. Infected, direct contact and respiratory contact ferrets in the ma-Ca/04-infected and Ca/04-infected groups shed similar amounts of virus over time (Fig 2A, B). Peak virus titers were slightly higher in the ma-Ca/04 group however the differences were not statistically significant (not shown). Likewise, the transmission kinetics: onset of sneezing, peaks in body temperature, and body weight losses were similar between the two groups (Table S1 and Figure S2) and to our previous studies with Ca/04 [23]. The exception was one ferret inoculated with the ma-Ca/04 showed significant diarrhea and body weight loss and was humanely sacrificed at 6 dpi (Table S1). Replication and virus distribution in different tissues demonstrated that titers in the nasal turbinates of ferrets infected with ma-Ca/04 increased 10 fold over the Ca/04 group (Fig 2C). Furthermore, the virus was isolated from the olfactory bulb in one out of two ma-Ca/04 infected ferrets, whereas no virus was detected in the olfactory bulb from Ca/04-infected ferrets (Fig 2C). There was no obvious difference in titers from lung and trachea, and virus could not be isolated from the brain in either group (Fig 2C). These observations suggest that the ma-Ca/04 maintains its infectivity and transmissibility for ferrets at levels comparable to those observed with the Ca/04 virus.

### Five amino acid substitutions responsible for the mouse- adaptation of Ca/04

**Figure 3:**
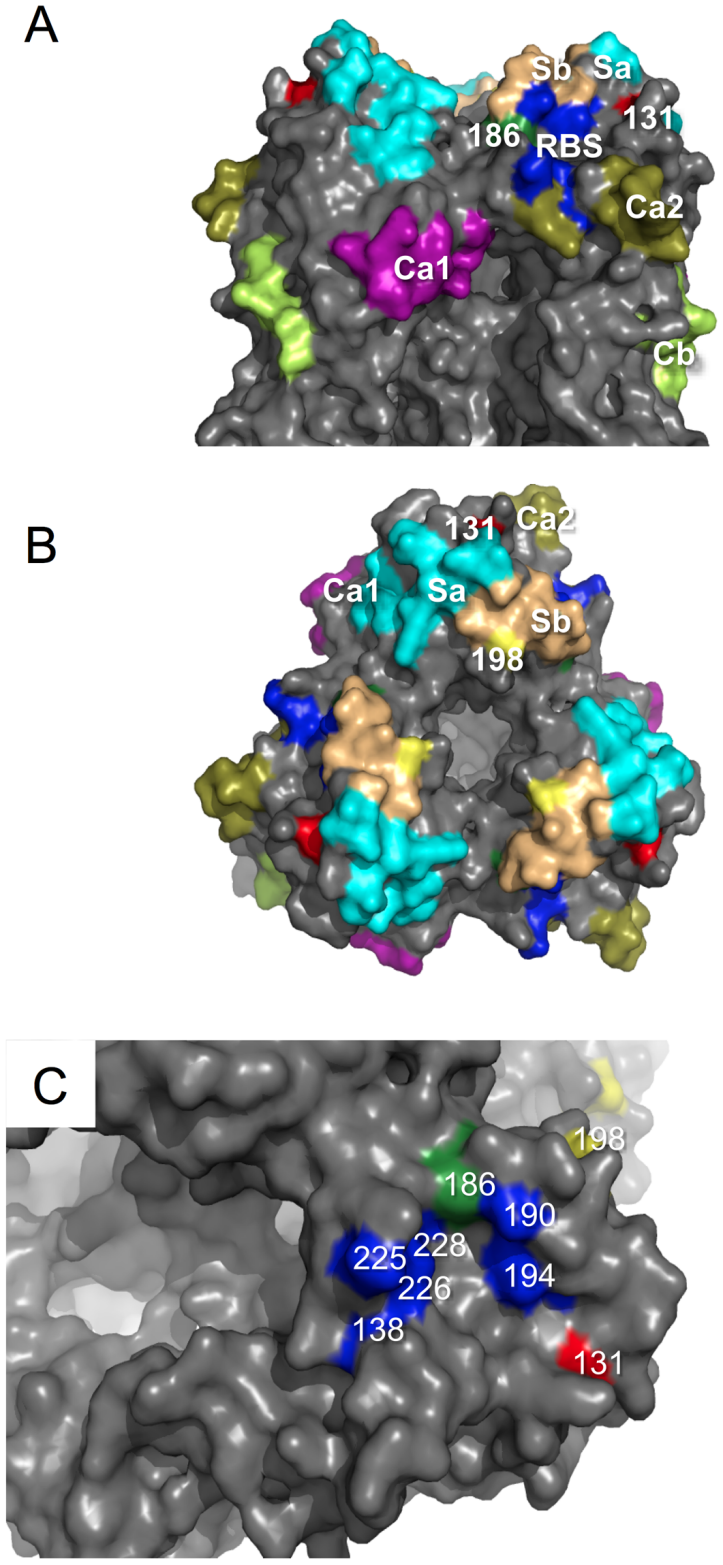
Structural analysis of amino acid mutations on the HA of ma-Ca/04. A) Side view of the H1 HA trimeric structure based on A/South Carolina/1/1918 (H1N1) (PDB code: 1RUZ) [24] showing the 5 major antigenic sites [29] on the globular head of the molecule with respect to amino acid changes between the ma-Ca/04 and Ca/04 viruses. RBS, receptor-binding site (dark blue); 131, D131E mutation (red); 186, S186P mutation (green); Sa, antigenic site A (cyan); Sb, antigenic site B (light orange); Ca1, antigenic site C2a (purple); Ca2, antigenic site C2a (olive); Cb, antigenic site Cb (lemon). B) Top view of H1 HA trimer as described in A); 198, A198E mutation (yellow) present on Sb. C) Close-up of the RBS in one of the HA monomers, highlighting amino acids involved in receptor binding (138, 190, 194, 225, 226, and 228, in blue) with respect to amino acid mutations between ma-Ca/04 and Ca/04 (D131E, red; S186P, green - part of the RBS -; and A198E, yellow). Structures generated using MacPymol (DeLano Scientific).

To identify the molecular markers responsible for the virulence of ma-Ca/04 in Balb/c mice, the viral genome was sequenced and compared to Ca/04, as well as other H1N1pdm influenza strains. Sequence analysis revealed that only five amino acid substitutions had occurred during the adaptation of Ca/04: 3 in HA (D131E, S186P and A198E, based on the mature 1918 virus HA protein sequence [24], [25]), 1 in PA (E298K) and 1 in NP (D101G) (Fig 3 and Table 2). There were no amino acid mutations found in the PB2, PB1, NA, M and NS genes. The small number of mutations observed may be related to the low passage in mouse lungs as suggested previously [26], [27]. The D131E mutation sits outside the major antigenic site Sa and the receptor-binding site (RBS), potentially modulating access to the sialic acid receptor (Fig 3A, B, and C). The S186P mutation occurs within the RBS and so this mutation would have more of a role modulating access to the sialic acid than position 131. The S186P mutation overlaps also with antigenic site Sb (Fig 3A and C). Likewise, the A198E mutation sits within the edge of Sb, and is under immunological pressure (Fig 3B and C) [28], [29]. In fact, E198 is common in seasonal H1N1 strains [28], [29]. Further comparative sequence analysis showed that some current pandemic strains share the same D131E or S186P mutation in HA. As listed in Table 2, the amino acid at position 131 is E in strains A/Hiroshima/220/2009(H1N1) and A/Singapore/TLL52/2009(H1N1), and a mixture of D and E in strains A/New York/09/2009 (H1N1), A/New York/12/2009 (H1N1), A/New York/39/2009 (H1N1) and A/Ohio/07/2009 (H1N1). And the amino acid at position 186 is P in strains A/Ankara/17/2009 (H1N1), A/California/VRDL7/2009 (H1N1), A/Ontario/25913/2009 (H1N1) and A/Swine/NC/19646/2010 (H1N1), and a mixture of S and P in strains A/Kansas/03/2009 (H1N1) and A/Ontario/10016/2009 (H1N1). Likewise, the D101G mutation in the NP gene is found in the A/Mexico/4108/2009 (H1N1) strain, and the E298K mutation in the PA gene is found in the strain A/Korea/01/2009 (H1N1). These comparisons suggest that our adaptation scheme did not select for mutations unique to virus adaptation in mice. Instead, it implies that the Ca/04 may contain a mixed virus population, which was selected out in DBA/J2 mice. It is worth noting that two mutations in HA (D131E and S186P) are also highly conserved in the 1918 pandemic H1N1 strains sequenced to date (Table 2). It is tempting to speculate that amino acids at positions 131 and 186 are potential virulent determinants in H1N1 influenza viruses.

**Table 2:** Comparison of amino acid differences between Ca/04 and ma-Ca/04 viruses and other H1N1 viruses.

Virus	HA			PA	NP
	131	186	198	298	101
Ca/04	D	S	A	E	D
ma-Ca/04	**E**	**P**	**E/A**	**K**	**G**
A/Netherlands/602/2009 (H1N1)	D	S	A	E	D
A/New York/18/2009 (H1N1)	D	S	A	E	D
A/Hiroshima/220/2009 (H1N1)	**E**	S	A	E	D
A/Singapore/TLL52/2009 (H1N1)	**E**	S	A	E	D
A/Ohio/07/2009 (H1N1)	**D/E**	S	A	E	D
A/New York/09/2009 (H1N1)	**D/E**	S	A	E	D
A/New York/12/2009 (H1N1)	**D/E**	S	A	E	D
A/New York/39/2009 (H1N1)	**D/E**	S	A	E	D
A/Mexico/4108/2009 (H1N1)	D	S	A	E	**G**
A/Korea/01/2009 (H1N1)	D	S	A	**K**	D
A/Ankara/17/2009 (H1N1)	D	**P**	A	-	-
A/Kansas/03/2009 (H1N1)	D	**S/P**	A	E	D
A/California/VRDL7/2009 (H1N1)	D	**P**	A	E	D
A/Ontario/10016/2009 (H1N1)	D	**S/P**	A	E	D
A/Ontario/25913/2009 (H1N1)	D	**P**	A	E	D
A/Swine/NC/19646/2010 (H1N1)*	D	**P**	A	**-**	-
A/Brevig Mission/1/1918 (H1N1)	**E**	**P**	A	E	D
A/New York/1/1918 (H1N1)	**E**	**P**	A	-	-
A/South Carolina/1/1918 (H1N1)	**E**	**P**	A	-	-
A/London/1/1918 (H1N1)	**E**	**P**	A	-	-
A/London/1/1919 (H1N1)	**E**	**P**	A	-	-

(-) No sequence information.

*2009 pandemic H1N1 strain isolated from swine population.

### Mutations in ma-Ca/04 PA and NP modulate polymerase activity and growth kinetics

**Figure 4:**
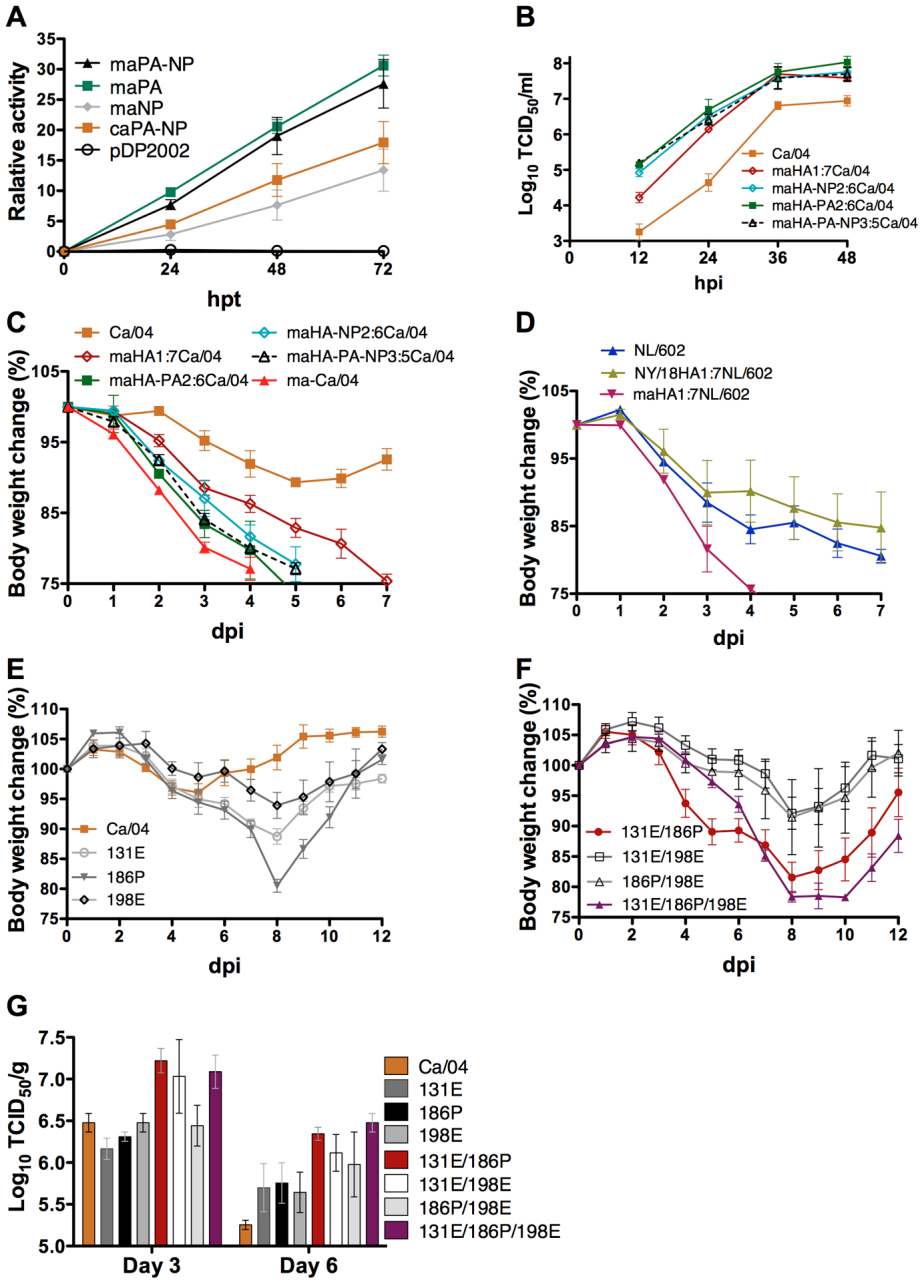
Effects of amino acid mutations for virulence of ma-Ca/04. A) Polymerase activity measured with mini-genome assay. Each transfection consisted of 6 plasmids encoding a minigenome influenza replicon (Gluc flanked by influenza NS gene untranslated regions), PB1, PB2, PA, NP encoding plasmids from Ca/04 (or ma-Ca/04 as indicated) and SEAP (used to normalize transfection efficiency). Relative activity calculated as the fold difference in the ratio of Luc/SEAP activity as described in materials and methods. Data corresponds to three independent experiments with samples run in duplicates. The maPA corresponds to transfection of the ma-Ca/04 PA gene, the maPA-NP corresponds to transfection of the ma-Ca/04 PA and ma-Ca/04 NP genes, the maNP corresponds to transfection of the ma-Ca/04 NP gene, the caPA-NP corresponds to transfection of the Ca/04 PA and Ca/04 NP genes, and pDP2002 corresponds to empty vector control, respectively, in the minigenome assay. B) Growth kinetics of recombinants of Ca/04 carrying either HA (maHA1:7Ca/04), HA and PA (maHA-PA2:6Ca/04), HA and NP (maHA-NP2:6Ca/04), or HA, PA, and NP (maHA-PA-NP3:5Ca/04) from ma-Ca/04. Viruses were inoculated at a multiplicity of infection of 0.001. Supernatants were collected at the indicated time points and titrated in MDCK cells by TCID_50_. C) % body weight over time in Balb/c mice (n = 3) infected with viruses produced in B) (1.2×10^5^ TCID_50_/mouse). D) % body weight over time in Balb/c mice (n = 3) infected with recombinant viruses of NL/602 carrying either the wt HA from NL/602 (NL/602), ma-Ca/04 HA (maHA1:7NL/602) or NY/18 HA (8.0×10^4^ TCID_50_/mouse). E) and F) % body weight over time in Balb/c mice (n = 5) infected with recombinants carrying single, double or triple mutations in HA in the backbone of Ca/04 (8.0×10^4^ TCID_50_/mouse). G) Mice (n = 6/virus) inoculated intranasally with mutants produced in E) (8.0×10^4^ TCID_50_/mouse). Lungs from infected mice (n = 3/time point) were collected at 3 and 6 dpi, homogenized and virus titers measured in MDCK cells by TCID_50_. Results are expressed as log_10_ TCID_50_/gr of tissue.

Although the ma-Ca/04 point mutations in ma-PA (E298K) and ma-NP (D101G) are present in naturally occurring H1N1pdm strains, they are not reflected in the consensus sequences for these two viral proteins. Therefore, we wanted to evaluate if such mutations affect polymerase activity. We assessed activity, using an influenza minigenome assay consisting of a secreted luciferase influenza replicon and plasmids encoding the polymerase complex and NP - as described in materials and methods. Polymerase activity was tested at different times post-transfection. The ma-PA and ma-NP were tested individually, as well as in combination, in the Ca/04 background, and compared to Ca/04 (Fig 4A). The ma-PA E298K mutation increased polymerase activity 2.0-fold compared to the wt PA from 24 h to 72 hours post-transfection (hpt), whereas the ma-NP D101G mutation (when tested without the ma-PA) reduced polymerase activity by ∼0.9 fold compared to the wt NP. The combined effect of ma-PA K298 and ma-NP G101 resulted in fold increases of ∼1.6 at 24 hpt, and ∼1.8, at 72 hpt over the wt, consistent with a ∼2 log_10_ increase in growth kinetics for viruses carrying the ma-PA or the ma-PA and ma-NP mutations (in the context of the ma-Ca/04 HA gene) compared to the Ca/04 virus (Fig 4B).

### Mutations in HA, PA and NP contribute to the virulence of ma-Ca/04

To determine the role of each ma-Ca/04 mutation in the virulence phenotype, the HA, PA and NP genes from the ma-Ca/04 were cloned in a reverse genetics vector as previously described [30]. Four recombinant viruses (Table 1) were generated using the Ca/04 backbone. Pathogenesis studies in Balb/c mice indicated that the three mutations in HA caused significant morbidity and weight loss (maHA1:7Ca/04) however, the double mutants maHA-PA2:6Ca/04 and maHA-NP2:6Ca/04 were more virulent than maHA1:7Ca/04. The triple mutant, maHA-PA-NP3:5Ca/04, was the most virulent recombinant tested (Fig 4C), indicating all five mutations appear necessary to match the phenotype observed with ma-Ca/04. Interestingly, MLD_50_ studies demonstrated that mutations in HA alone (maHA1:7Ca/04) contributed to a moderate increase in lethality; however, lethality increased in the context of the ma-PA and ma-NP. These results indicate a synergistic effect for the mutations in HA, PA and NP in ma-Ca/04 for virulence (Table 1).

In order to confirm whether the virulent phenotype imparted by mutations in the HA of ma-Ca/04, could modulate the virulence of related H1N1pdm viruses, we generated additional viruses in the backbone of A/Netherlands/602/09 (H1N1) –NL/602- (Table 1 and Fig 4D). We observed that the MLD_50_ of wt NL/602 went from 6.0 to 3.4 when provided the maHA gene; maHA1:7NL/602 provided a MLD_50_ similar to the ma-Ca/04 virus. Previous reports have shown that the NL/602 is lethal to Balb/c mice without prior adaptation (Fouchier R., pers communication). Our results are consistent with this observation, suggesting that the NL/602 virus contains a gene constellation more virulent than Ca/04. There are 8 amino acid differences between Ca/04 and NL/602: 3 in HA (P87S, T200A and I324V), 1 in PA (M581L), and 1 in NP (P224S). The T200A mutation is close to the A198E mutation found in ma-Ca/04 and just outside of antigenic site B [29]. The I324V lies outside the HA cleavage site, but its effects on cleavage remains to be determined. It also unknown whether the amino acid differences in PA and NP are responsible for the increased virulence of NL/602 in mice compared to the Ca/04. However, the virulence of NL/602 for mice can be greatly enhanced by incorporating the HA gene from the ma-Ca/04 virus. The mice inoculated with 8.0×10^4^TCID_50_ of maHA1:7NL/602 succumbed to the infection by 5 dpi (Fig. 4D). To further determine the effect of the HA mutations in virulence, we created a recombinant virus carrying the HA gene from A/New York/18/2009 (H1N1) (NY/18) in the backbone of NL/602 (NY/18HA:7NL/602). The NY/18 virus contains D131, S186 and A198 in the HA, the same amino acids found in Ca/04 at those positions. The Ca/04 and NY/18 strains differ at HA positions (P87S, T200A, S206T and I324V). In this regard, the NL602 and NY/18 differ only at position S206T. As expected the MLD_50_ of NY/18HA1:7NL/602 is >6.0 (Table 1). Mice infected with 8.0×10^4^TCID_50_ of either NL/602 or NY/18HA1:7NL/602 lost roughly 20% and 15%, respectively, of their body weight within the first 7 dpi but eventually recovered (Fig 4D). These studies are consistent with the notion that mutations in the HA of mouse-adapted viruses can modulate virulence [26], [27].

### 131E/186P combination is the major contributor to virulence imparted by ma-HA

Since the D131E and S186P mutations in the HA of ma-Ca/04 resemble amino acids present on the HA of the 1918 pandemic viruses (Table 2), we hypothesize that these mutations were responsible for the increased virulence of ma-Ca/04. To pinpoint the contribution of each amino acid mutation in HA to the virulence of ma-Ca/04, single and double mutants were generated using the remaining 7 genes from Ca/04 (1:7 recombinants). Balb/c mice (n = 5) infected with 8.0×10^4^TCID_50_ of single mutant 186P, 131E, and 198E viruses showed average maximum bodyweight losses of 19%, 11% and 6%, respectively. In contrast, mice infected with 8.0×10^4^TCID_50_ of Ca/04 lost only 3.9% of body weight (Fig 4E). These results demonstrate major contributions to virulence for amino acids at positions 131 and 186 in HA. Lung virus titers of mice inoculated with single mutant 186P, 131E, and 198E viruses were similar to those for Ca/04 at 3 dpi (Fig 4G), and were approximately 0.4_,_ 0.5, and 0.4 log_10_ TCID_50_ higher, respectively, than for Ca/04 at 6 dpi (Fig 4G). Lung virus titers were similar for the three single point mutants; however 186P and 131E viruses caused more morbidity than 198E virus indicating that virus replication alone cannot account for differences in virulence.

Mice infected with either double (131E/186P) or triple (131E/186P/198E) HA mutant virus lost weight rapidly. The average maximum body weight loss of mice infected with 131E/186P or 131E/186P/198E was 18% and 22%, respectively (Fig 4F). However, mice infected with mutants 131E/198E or 186P/198E lost 8% and 9% of their body weight, respectively (Fig 4F). These results suggest that the 131E/186P combination is the major contributor to virulence imparted by HA with the 198E mutation adding virulence only when present with the 131E/186P mutations. Lung virus titers for 131E/186P, 131E/198E and 131E/186P/198E were approximately 0.7, 0.6, and 0.6 log_10_ TCID_50_, respectively, higher than for Ca/04 at 3 dpi (Fig 4G). Additionally, lung virus titers for the 131E/186P, 131E/198E, 186P/198E, and 131E/186P/198E mutants were approximately 1.1, 0.9, 0.7 and 1.2 log_10_ TCID_50_ higher than for Ca/04 at 6 dpi (Fig 4G). Thus, these single and double mutants clearly demonstrate that the D131E and S186P mutations, alone and in combination, can increase the virulence of the pandemic H1N1 virus in Balb/c mice.

### Mutations found in ma-HA cause minor antigenic changes

Given that positions 131, 186 and 198 in HA are located within or in close proximity to major antigenic sites and that 186 is within the RBS pocket (Fig 3), it was anticipated that these mutations could modulate antigenicity and receptor binding. Therefore, we used four ferret sera and two monoclonal antibodies (mAbs) raised against the H1N1pdm virus to evaluate the antigenic profile of ma-Ca/04 and compared it to other H1N1pdm viruses (Table 3 and data not shown). The four ferret sera showed a distinct HI profile dependent on the HA mutation. These sera had the lowest HI titer when HA protein carried 131E and/or 186P mutations (Table 3). The 131E/186P double mutation resulted in 7.4-fold lower HI titer than those obtained with the Ca/04 virus generated by reverse genetics. The 131E and 186P single mutants also displayed 4.5-fold and 3.9-fold lower HI titers, respectively, compared to the Ca/04 virus. In contrast, the single 198E mutation resulted in the highest HI titer (1.8-fold higher than with Ca/04). The HI profiles from 131E, 186P, 198E and 131E/186P mutant viruses were significantly different (p<0.01) than the one obtained for Ca/04. However, the HI titers from the double mutants, 131E/198E, 186P/198E, and the triple mutant, 131E/186P/198E, showed no statistical differences with the HI titer obtained for Ca/04 (p>0.05). Interestingly, one of the two monoclonal antibodies, mAb 5H7, had a different HI profile dependent on the HA mutations, mAb 3B2, showed no differences in the HA profile, regardless of the mutant used. The mAb 5H7 had the highest HI titers when the HA protein carried the 186P mutation, whether alone as the single mutant or in concert with the 131E and/or 131E/198E viruses. These viruses resulted in 8-fold higher titers than those obtained with Ca/04. The 131E single mutant also showed a 4-fold higher HI profile than the Ca/04. In contrast, the 198E single mutant had an HI titer 4-fold lower than the Ca/04 virus. It is also worth noting that the 198E mutation appears to affect HI titers using either the ferret polyclonal sera or the mAb 5H7 in the context of double mutations, either in association with 131E or 198P. Coincidentally, the HI profile analysis for these HA mutants follows the pattern of virulence of ma-Ca/04. Mutations at positions 131 and 186 had the highest impact by altering the antigenic make up of the molecule and its virulence.

**Table 3:** HI profile of Ca/04-derived HA mutant viruses using ferret polyclonal sera and two mouse monoclonal antibodies developed against Ca/04.

Antibody	Virus							
	Ca/04	131E	186P	198E	131E/186P	131E/198E	186P/198E	131E/186P/198E
**Ferret sera** a	3200426732003200	4001067667800	10671067800667	6400853353335333	400667400400	6400640042673200	5333640042673200	2133266716001600
**mAb 5H7**	1024	4096	8192	256	8192	1024	4096	8192
**mAb 3B2**	8192	8192	8192	8192	8192	8192	8192	8192

a , HI titers using 4 different ferret sera raised against Ca/04 obtained by reverse genetics.

### Host range implications of 131E and 186P mutations in H1N1 viruses

We have shown that the D131E and S186P mutations increased the virulence for mice and conferred minor antigenic changes to the Ca/04 H1N1pdm virus. To determine the prevalence of such mutations we evaluated the host adaptation and/or host selection profile of H1 HA proteins from avian, swine, and human influenza viruses. From 1930 to 1998, 100% of H1 HAs from North American swine H1N1 isolates carried 131E, whereas 97% of them had 186P (including the prototype strain A/swine/Iowa/15/1930, SW30). North American swine H1N1 viruses started to carry the 131D and 186S mutations from 1999 onward. Analyses of swine H1 influenza sequences in Genbank indicate that from 1999 to 2009, North American swine H1N1 contain 81% 131D and 67% 186S. This data indicates a dominant presence of 131D and 186S in the recent North American swine H1N1 viruses, which have resulted through evolution in the swine population. In contrast, 100% of avian H1N1 influenza viruses, isolates collected from 1976 to 2009, carry 131E and 98% encode for 186P. Only one isolate, A/quail/Nanchang/12-340/2000 (H1N1), possesses 186S. Interestingly, up until 1919, 100% of human H1N1 strains contained 131E and 186P (1918-like H1N1 strains). Unfortunately there is a hiatus of influenza sequence information from 1920 to 1932; however, from 1933 to 1997, only 6% of the human H1 isolates contained 186P. The S186P mutation was prominent from 1998 to 2000 (86%) and now dominates since 2000 (99% isolates). In human strains, position 131 has transitioned from E131 to N131, and then to practically 100% of T131. With the exception of prior swine influenza strains that sporadically crossed to humans, no human H1N1 strain carried D131 until the introduction of H1N1pdm viruses. Since the 131E and 186P mutations were present in the 1918 H1N1 viruses of avian origin, we refer to these as avian-type mutations whereas the 131D and 186S mutations are labeled as swine-like mutations. Our analysis suggests that in humans, H1 influenza viruses have shown predilection for the 131E, 186P avian-type combination, which could lead to viruses with altered virulence phenotypes.

## Discussion

The 2009 pandemic H1N1 prototypical strain, A/California/04/2009 (Ca/04), was isolated from a pediatric patient with uncomplicated, upper respiratory tract illness [31]. In general, infections with the 2009 H1N1 pdm virus have been mostly mild. Mortality is usually associated with at-risk groups, including those with chronic lung and heart conditions, obesity and, most strikingly, pregnancy [32], [33]. However, some deaths have occurred in individuals with no known underlying conditions and recovery of some critically ill children have resulted in neurological sequeleae [32], [33], [34], [35], [36]. Previous reports indicate that Ca/04 is also mild in Balb/c mice (MLD_50_>6 log_10_ PFU), unlike the 1918 pandemic influenza strains [31]. In this study, we confirmed the mild phenotype in Balb/c mice and found that Ca/04 H1N1 causes significant morbidity/mortality only after inoculation with a high virus dose (MLD_50_>6 log_10_ TCID_50_). In contrast, the Ca/04 virus was lethal for DBA/J2 mice, a mouse strain shown to be more susceptible to influenza [17], [18]. Surprisingly, the virus isolated from the lungs of the DBA/J2 mice was lethal for Balb/c mice. This lethality was maintained in Balb/c mice after subsequent passage of the virus obtained from the DBA/J2 mice lungs to MDCK cells. The lethal virus – ma-Ca/04 – was sequenced and compared to the parental Ca/04. Five amino acid differences were found between the ma-Ca/04 and Ca/04 viruses, which were responsible for improving virus replication and virulence. Mutations in PA (E298K) and NP (D101G) modulated polymerase activity and contributed to virulence, while mutations in HA (D131E, S186P, A198E) had the highest impact on virulence for mice. These mutations not only affected the virulence of the H1N1pdm virus in the context of the Ca/04 background, but also the related NL/602 pdm strain. These results suggest a dominant effect of HA mutations, particularly those at positions D131E and S186P. These same mutations also affected the antigenicity of HA as indicated by the differential HI profile using polyclonal and monoclonal antibodies. Altering receptor-binding activities has been proposed as a mechanism that also alters antigenicity [37]. Changes in the HI profile suggest structural changes that are consistent with this notion. In this regard, it is worth noting the contribution of the S186P mutation, which is under immunological pressure (Site b), but which also appears to be under selection pressure in humans. The conservation of P186 in current human H1N1 influenza viruses, prior to the H1N1pdm viruses, is indeed remarkable. Furthermore, sequence analysis shows the presence of P186 in several 2009 H1N1 pdm viruses, occasionally as a mixed population with S186 (Table 2). More recently, the sequence of the HA gene of a 2009 H1N1 pdm isolated from swine was shown to contain P186. Since these changes in HA are either close (131) or within (186) the RBS site, we are tempted to speculate that they modulate virulence by altering binding to sialic acids receptors. It is important to mention that sera from ferrets infected with recent seasonal H1N1 strains reacted poorly against the ma-Ca/04 and Ca/04 viruses (not shown), and although ferret sera against reverse genetics Ca/04 virus showed no significantly distinguishable HI activities against the 131E/186P/198E triple mutant, the HI profiles of the single mutants, 131E, 186P, and 198E, and the double mutant, 131E/186P, were significantly different from the Ca/04 virus (p<0.01). Thus, it remains to be seen what type of impact a virus like ma-Ca/04 would have on the human population. Those previously vaccinated and/or infected with the H1N1pdm strain should have significant cross-neutralizing antibodies.

Sequence analysis revealed that mutations found in ma-Ca/04 are also found scattered throughout other H1N1pdm strains, although the combination of E131, P186, and E198 in HA, K298 in PA and G101 in NP were unique to ma-Ca/04. The quick selection of ma-Ca/04 in mice suggests that a virus with this constellation of amino acids might have been already present in Ca/04, likely as minor quasispecies. In this regard, it is important to consider the limited understanding of influenza quasispecies for their potential contribution to host range and evasion of immune surveillance, despite the great wealth of knowledge stemming from the current influenza genome sequencing efforts. Selection of the S186P mutation was obtained independently by plaque purification of the Ca/04 virus in MDCK cells, indicating that a mixed population is likely to exist (not shown). However, we did not isolate the other four mutations observed after a single passage of Ca/04 in DBA/J2 mice. It must be noted that plaque selection may not necessarily be the best method to look for quasispecies as plaque purification itself is simply another selection method for a virus population that can create plaques. Sequence analysis of field isolates indicate mixed populations at positions 131 and 186, which we would like to speculate may be due to host pressure. In this regard, the ideal approach for understanding influenza quasispecies would be to rely on deep sequencing of viruses present in the original swab sample. Such an approach is beyond the scope of the present report. We must also note that we did not attempt to adapt the virus directly in Balb/c mice, which could have resulted in a different set of mouse-adapted mutations, unlike those described in this report. In fact, this is indeed the case as reported by Jacobs et al. (pers. communication). At the time of writing of this report, Ilyushina et al reported the adaptation of two H1N1pdm viruses, including Ca/04, in Balb/c mice (J. Virol. 2010, in press). Interestingly, nine amino acid positions were under selective pressure including four in the ribonucleoprotein (RNP) complex (PB2 E158G/A, PA L295P, NP D101G, and NP H289Y) and five in the HA glycoprotein (K119N, G155E, S183P, R221K, and D222G – H1 HA numbering). Interestingly, NP D101G and HA S183P (186 in H3 numbering) were also found in our study. In this latter report, however, the effects for virulence in mice of amino acid mutations on the HA were not analyzed. Our report clearly shows a significant impact on virulence in mice provided by the 186P mutation, particularly in the context of the 131E mutation.

Our results demonstrate the association between minor antigenic changes involved in receptor binding and modulation of virulence of influenza strains. The seasonality of influenza is thought to occur, at least in part, due to the virus's ability to evade immune surveillance through antigenic drift. Less is known about how antigenic changes lead to changes in virulence. In this report we show that two amino acid mutations in the H1N1pdm HA, at positions 131 and 186, can modulate virulence for mice. Based on our extensive sequence analysis, we would like to speculate that these amino acids are also responsible, among other potential factors, for the mild presentation of the H1N1pdm in humans. We consider the tracking of changes like these on the HA molecule to be important to predict the evolutionary virulence of the new H1N1pdm viruses.

## Materials and Methods

### Viruses and cells

A/California/04/09 (H1N1) (Ca/04) and A/New York/18/09 (H1N1) (NY/18) were kindly provided by the Centers for Disease Control and Prevention (CDC), Atlanta, Georgia. These viruses were provided as passage 2 viruses in MDCK cells. Passage 3 viruses were generated and stocks prepared and maintained at −70°C until use. Alternatively, stocks were prepared using viruses rescued by reverse genetics as indicated below. The A/Netherlands/602/09 has been previously described and was obtained by reverse genetics using plasmids kindly provided by Ron Fouchier, Erasmus Medical Center, The Netherlands [38]. Viruses were titrated in MDCK (Madin-Darby canine kidney) cells to determine the TCID_50_ by the Reed and Muench method [39]. MDCK cells were maintained in Modified Eagle's medium (MEM) (Sigma-Aldrich, St. Louis, MO) containing 5% fetal bovine serum (FBS) (Sigma-Aldrich). 293-T human embryonic kidney cells were cultured in Opti-MEM I (GIBCO, Grand Island, NY) containing 5% FBS.

### Mouse studies

Five-week-old female mice (Balb/c and DBA/J2) (Charles River Laboratories) were anaesthetized with isofluorane before intranasal inoculation with 50 µl virus suspension. In an initial evaluation, DBA/J2 and Balb/c mice (n = 4) were infected intranasally with the Ca/04 virus (5.4×10^5^ TCID_50_). Body weight changes and survival were recorded daily. Mice presenting ≥25% body weight loss were humanely euthanized and counted as dead. For adaptation studies lungs were collected from DBA/J2 mice, which succumbed to infection with Ca/04 (n = 2). Lungs were homogenized in PBS with antibiotics. After centrifugation at 6,000 rpm for 10 min, 50 µl of supernatant from the homogenate was passaged to naïve Balb/c mice (n = 3). Lungs from these infected Balb/c mice were then homogenized and inoculated into MDCK cells to prepare a virus stock. The 50% mouse lethal dose (MLD_50_) was calculated using different virus doses in Balb/c and DBA mice (n = 3/per virus dose). Body weight and survival were recorded daily until 14 dpi. To evaluate the replication tropism of selected H1N1 pdm viruses in mice, lungs were collected at 3 and 5 dpi and titrated in MDCK cells. For histopathology analysis, mouse lungs collected at 3 dpi were fixed in 10% formalin, embedded in paraffin, sectioned and stained with hematoxylin and eosin (H&E). In order to evaluate differences in virulence of Ca/04 compared to related ma-Ca/04-derived mutants, the virus doses were adjusted to 8.0×10^4^ TCID_50_/mouse.

### Ferret studies

The experimental design to study transmission of influenza in ferrets has been previously described [40]. Briefly, two ferrets (1/cage) were lightly anesthetized with ketamine (20 mg/kg) and xylazine (1 mg/kg) via an intramuscular injection and were inoculated intranasally with 10^6^ TCID_50_ of virus in PBS, 250 µl per nostril. At 24 hpi, two naïve ferrets (1/cage) were introduced in direct contact with the infected ferret. A second ferret (1/group) was introduced as a respiratory contact separated from the infected and direct contact by a wire mesh wall with no possibility of physical contact. To monitor virus shedding and transmission nasal washes were collected daily and titrated in MDCK cells. Virus replication and tissue distribution were evaluated in groups of two ferrets/virus inoculated with 10^6^ TCID_50_ of either Ca/04 or ma-Ca/04, respectively. Ferrets were euthanized at 4 dpi. Brain, olfactory bulb, nasal turbinate, trachea, and lungs were collected, homogenized and titrated in MDCK cells as described [41].

### Ethics statement

Animal studies were conducted under ABSL-3 conditions approved by USDA and performed according to the protocol R-09-93 “Transmissibility of Influenza A Viruses” approved by the Institutional Animal Care and Use Committee of the University of Maryland.

### Sequence analysis

The vRNA and cDNA were prepared as previously described [30]. The eight fragments were amplified and sequenced using a combination of universal [42] and custom made primers (available upon request). Sequencing was performed using the Big Dye Terminator v3.1 Cycle Sequencing kit (Applied Biosystems, Foster City, CA) on a 3100 Genetic Analyzer (Applied Biosystems, Foster City, CA) according to the manufacturer's instructions.

### Generation of recombinant viruses by reverse genetics

The 8 gene segments of Ca/04 (passage 2 in MDCK cell) and ma-Ca/04 (stock, passage1 in MDCK cell) were amplified by RT-PCR and cloned in the bidirectional reverse genetics plasmid pDP2002 [30]. Mutations of interest in the HA gene were introduced using the QuickChange II site-directed mutagenesis kit (Stratagene, Inc., La Jolla, CA) according to manufacturer's protocols. The presence of each mutation was confirmed by sequencing. The recombinant viruses were recovered by transfection in co-cultured 293T and MDCK cells using eight plasmids as previously described [43]. Recovered viruses were sequenced to ensure the absence of unwanted mutations, titrated in MDCK cells and stocks were prepared and frozen at −70°C until use.

### Minigenome assay for viral polymerase activity

A model viral RNA (vRNA), consisting of the Gaussia Luciferase (GLuc) open reading frame flanked by the non-coding regions of the influenza NS segment was used to assess polymerase activity in a minigenome reconstitution assay. Briefly, 293T cells were seeded in 6-well plates and transfected with 1 µg of the reporter plasmid along with 1 µg of each of expression plasmids encoding PB2, PB1, PA and NP using the TransIT-LT1 (Mirus, Madison, WI) reagent following the recommendations of the manufacturer. In addition, the pCMV/SEAP plasmid, which encodes a secreted alkaline phosphatase gene, was co-transfected into the cells to normalize the transfection efficiency. At the indicated time points, supernatant from transfected cells were harvested and assayed for both luciferase and secreted alkaline phosphatase activities using either the BioLux Gaussia Luciferase Assay Kit (NEB, Ipswich, MA) or the Phospha-Light Secreted Alkaline Phosphatase Reporter Gene Assay System (A&D, Foster City, CA) according to the manufacturers' recommendation. Relative polymerase activity was calculated as the ratio of luciferase versus SEAP luminescence for three independent experiments with duplicate samples.

### Statistical analysis

Graphs were produced and statistical analysis performed using the Prism software package (GraphPad Software Inc., La Jolla, CA).

## Supporting Information

Table S1 Peak virus titers and clinical signs in groups of ferrets infected with either ma-Ca/04 or Ca/04 viruses.
(0.06 MB PDF)

Figure S1 Viral antigen distribution in lungs of H1N1pdm infected mice. Immunohistochemistry staining was performed using a biotin-conjugated monoclonal antibody prepared in our laboratory (3B2-Biotin) against the HA protein of the H1N1pdm virus and horseradish peroxidase-conjugated streptavidin. The viral antigen was visualized using AEC substrate set (BD Biosciences, California, USA). Lungs from Ca/04- and ma-Ca/04-infected Balb/c mice were collected at 3 dpi. Ca/04 virus antigen staining was detected in the bronchiolar lumen (A and B), however only rarely detected in the alveolar area (C). Infection with ma-Ca/04 resulted in the accumulation of extensive virus antigen positive cells in the bronchiolar lumen (D and E), and also focal positive staining could be detected in the alveolar area (F).
(3.75 MB TIF)

Figure S2 Clinical signs in ferrets infected with either Ca/04 or ma-Ca/04. A) Median of % body weight changes over time for ferrets infected with ma-Ca/04. Values were normalized using the average of body weights of inoculated ferrets at 0 (zero) dpi and represent the median values obtained from 4 groups of ferrets as described in Fig 2A. Symbols correspond to inoculated (infected, grey open squares), direct contact (orange triangles), and respiratory contact ferrets (purple squares). B) Median of % body weight changes over time for ferrets infected with Ca/04. Values normalized as in A) and represent the median values obtained from 2 groups of ferrets as described in Fig 2B. C) Body temperature changes (in Celsius) over time in ferrets infected with ma-Ca/04. Normal ferret temperature fluctuates between 37.2° to 40°C. Minor peaks in body temperature were observed for direct contact and respiratory contact ferrets between 1 to 4 dpi. Fever was observed for inoculated ferrets at 1 dpi. D) Body temperature changes (in Celsius) over time in ferrets infected with Ca/04. Minor peaks in body temperature were observed for the infected, direct contact and respiratory contact ferrets between 1 to 4 dpi.
(0.30 MB TIF)
