# Supplementary material for: Variations in the Hemagglutinin of the 2009 H1N1 Pandemic Virus: Potential for Strains with Altered Virulence Phenotype?
Source: PLoS Pathog. 2010 Oct 14;6(10):e1001145. doi: 10.1371/journal.ppat.1001145 (PMC2954835; doi:10.1371/journal.ppat.1001145)
Supplement: Table S1 — Peak virus titers and clinical signs in groups of ferrets infected with either ma-Ca/04 or Ca/04 viruses. (0.06 MB PDF) [file ppat.1001145.s001.pdf]

Table S1. Peak virus titers and clinical signs in groups of ferrets infected with either ma-Ca/04 or Ca/04 viruses.

Virus	ma-Ca/04[a]			Ca/04[b]		
Ferret group	Peak nasal wash titer[c]	Sneezing (Day of onset)[d]	Peak bodyweight loss (%)	Peak nasal wash titer	Sneezing (Day of onset)	Peak bodyweight loss (%)
Infected	6.7 ± 0.4	4/4 (3,4,4,4)	>20[e]; 3.4, 10.9, 6.6	6.7 ± 1.0	2/2 (4,5)	14.7, 5.3
Direct Contact	6.9 ± 0.5	4/4 (3,4,5,7)	10.7, 6.2, 0.0[f], 0.0	5.6 ± 0.6	2/2 (6,8)	8.8, 6.6
Respiratory Contact	6.6 ± 0.5	2/4 (8,11)	10.0, 3.6, 1.7, 8.8	5.5 ± 1.7	2/2 (9,9)	7.4, 9.3

[a] Data corresponds to two independent experiments with 2 groups each. Each group consisted of 1 inoculated ferret, 1 direct contact and 1 respiratory droplet contact as described [41].[b] Data correspond to one experiment with 2 groups as explained in (a).[c] Titers expressed in $log_{10}TCID_{50}$/ml of nasal wash. [d] Day of onset of sneezing calculated from 0 (zero) dpi. [e] Ferret sacrificed at 6 dpi. [f] Two ferrets in the direct contact groups gained weight.
