# Supplementary figures and images for: Variations in the Hemagglutinin of the 2009 H1N1 Pandemic Virus: Potential for Strains with Altered Virulence Phenotype?
Source: PLoS Pathog. 2010 Oct 14;6(10):e1001145. doi: 10.1371/journal.ppat.1001145 (PMC2954835; doi:10.1371/journal.ppat.1001145)

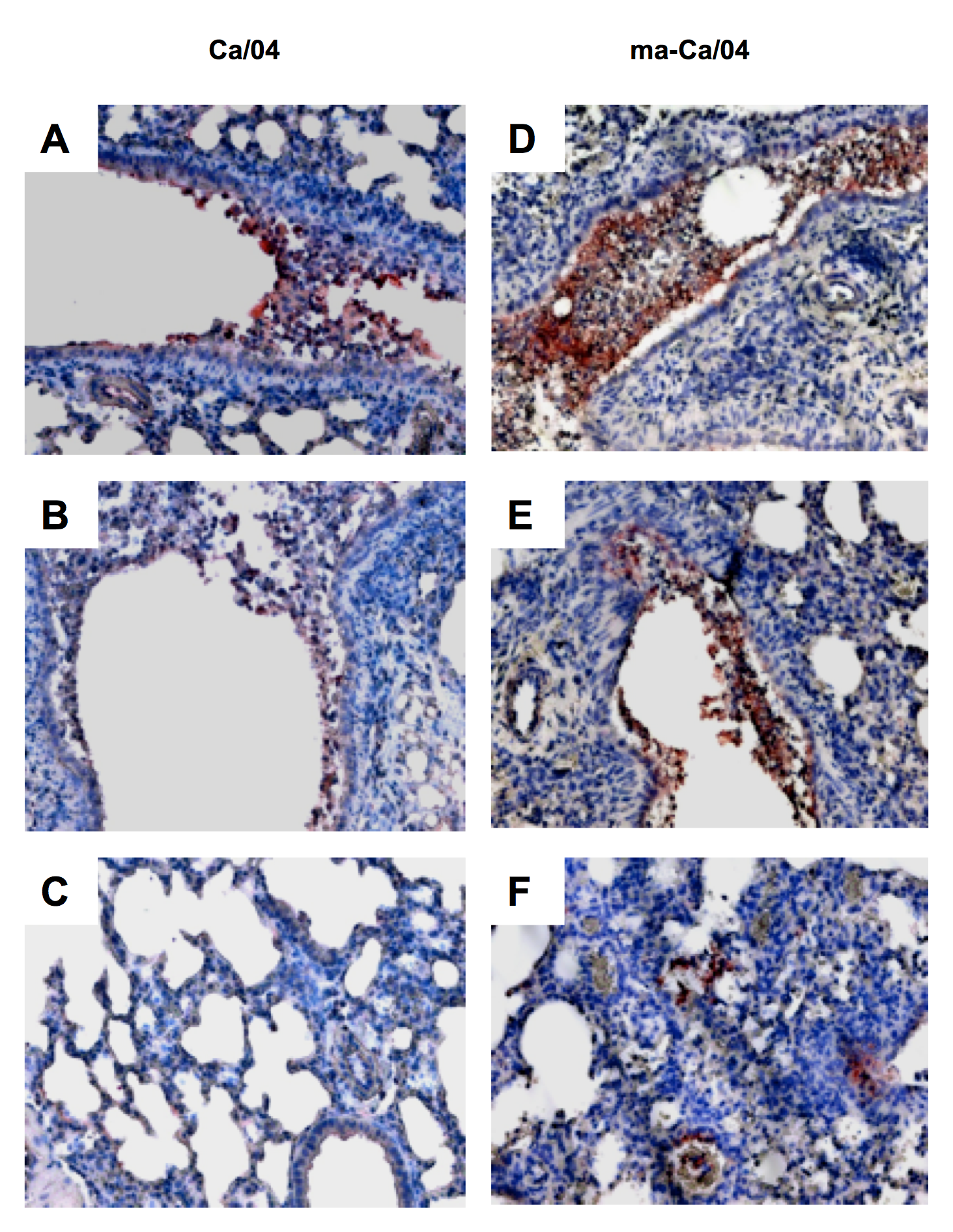

Supplement: Figure S1 — Viral antigen distribution in lungs of H1N1pdm infected mice. Immunohistochemistry staining was performed using a biotin-conjugated monoclonal antibody prepared in our laboratory (3B2-Biotin) against the HA protein of the H1N1pdm virus and horseradish peroxidase-conjugated streptavidin. The viral antigen was visualized using AEC substrate set (BD Biosciences, California, USA). Lungs from Ca/04- and ma-Ca/04-infected Balb/c mice were collected at 3 dpi. Ca/04 virus antigen staining was detected in the bronchiolar lumen (A and B), however only rarely detected in the alveolar area (C). Infection with ma-Ca/04 resulted in the accumulation of extensive virus antigen positive cells in the bronchiolar lumen (D and E), and also focal positive staining could be detected in the alveolar area (F). (3.75 MB TIF) [file ppat.1001145.s002.tif]

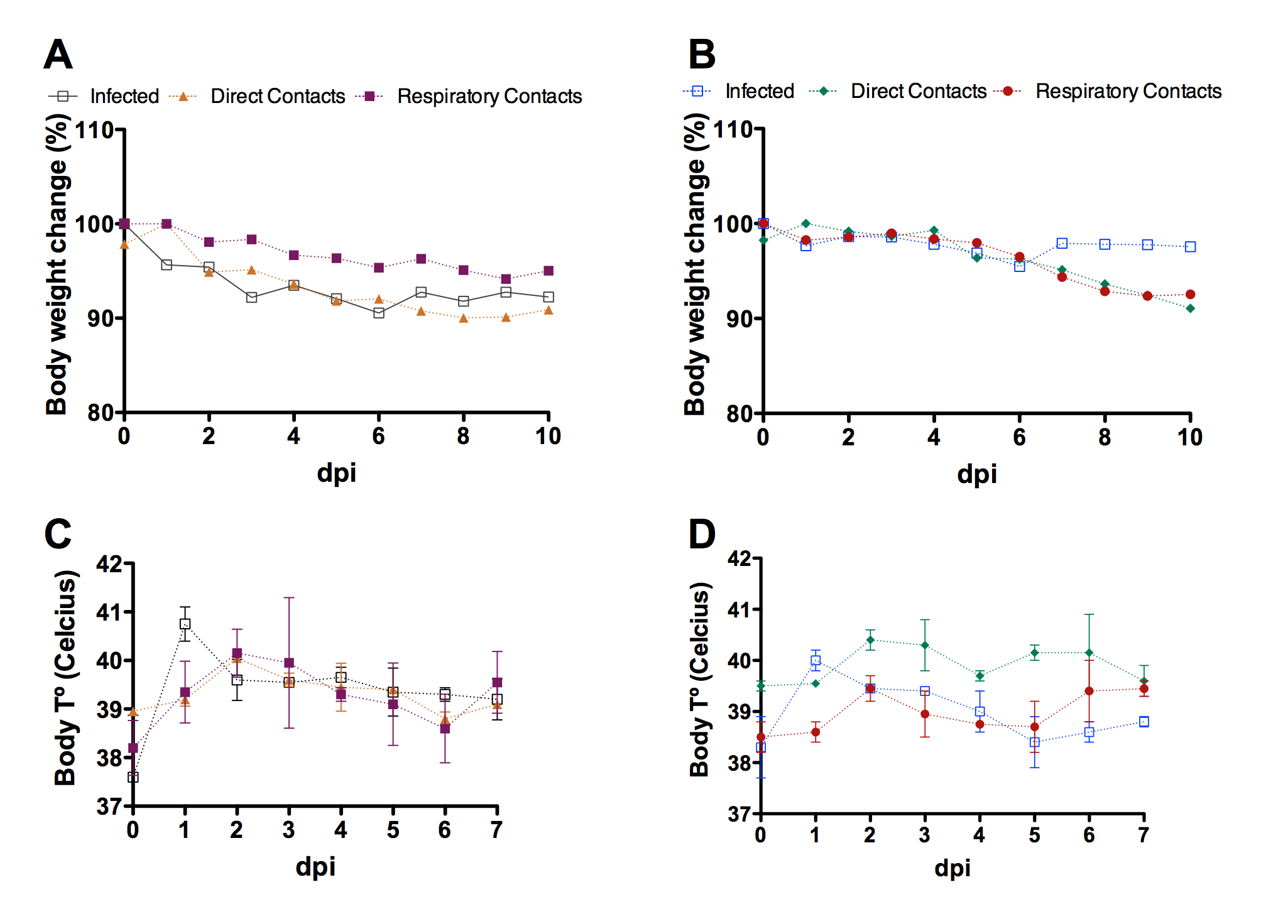

Supplement: Figure S2 — Clinical signs in ferrets infected with either Ca/04 or ma-Ca/04. A) Median of % body weight changes over time for ferrets infected with ma-Ca/04. Values were normalized using the average of body weights of inoculated ferrets at 0 (zero) dpi and represent the median values obtained from 4 groups of ferrets as described in Fig 2A. Symbols correspond to inoculated (infected, grey open squares), direct contact (orange triangles), and respiratory contact ferrets (purple squares). B) Median of % body weight changes over time for ferrets infected with Ca/04. Values normalized as in A) and represent the median values obtained from 2 groups of ferrets as described in Fig 2B. C) Body temperature changes (in Celsius) over time in ferrets infected with ma-Ca/04. Normal ferret temperature fluctuates between 37.2° to 40°C. Minor peaks in body temperature were observed for direct contact and respiratory contact ferrets between 1 to 4 dpi. Fever was observed for inoculated ferrets at 1 dpi. D) Body temperature changes (in Celsius) over time in ferrets infected with Ca/04. Minor peaks in body temperature were observed for the infected, direct contact and respiratory contact ferrets between 1 to 4 dpi. (0.30 MB TIF) [file ppat.1001145.s003.tif]
